# Orthogeriatric Care Following Hip Fracture: Improving Post-Operative Outcomes in an Aged Population

**DOI:** 10.3390/life14040503

**Published:** 2024-04-14

**Authors:** Sarah J. Mant, Chibuchi Amadi-Livingstone, Mohamed H. Ahmed, Maria Panourgia, Henry Owles, Oliver Pearce

**Affiliations:** 1 The Medical School, University of Buckingham, Buckingham MK18 1EG, UK; mant.sarahjane@gmail.com (S.J.M.); chibuchilivingstone@gmail.com (C.A.-L.); 2 Department of Medicine and HIV Metabolic Clinic, Milton Keynes University Hospital NHS Foundation Trust, Eaglestone, Milton Keynes MK6 5LD, UK; 3 Department of Geriatric Medicine, Milton Keynes University Hospital NHS Foundation Trust, Eaglestone, Milton Keynes MK6 5LD, UK; maria.panourgia@mkuh.nhs.uk (M.P.); henry.owles@mkuh.nhs.uk (H.O.); 4 Honorary senior lecturer. Faculty of Medicine and Health Sciences, University of Buckingham, Buckingham MK18 1EG, UK; 5 Department of Trauma and Orthopedics, Milton Keynes University Hospital NHS Foundation Trust, Eaglestone, Milton Keynes MK6 5LD, UK; oliver.pearce@mkuh.nhs.uk

**Keywords:** orthogeriatric, non-surgical site infection, post-operative care, cognitive impairment

## Abstract

Introduction: Hip fractures globally are associated with high levels of morbidity, mortality, and significant financial burden. This audit aimed to assess the impact of orthogeriatric liaison care on post-operative outcomes following surgical management of neck or femur fractures. Methods: Here, 258 patients who underwent hip fracture surgery over 1-year were included. Data were collected as an audit following the transition to an orthogeriatric liaison care model, involving regular orthogeriatric review (thrice weekly ward rounds, daily board rounds), superseding orthogeriatric review as requested. The audit is meant to assess the development of post-operative non-surgical site infection (NSSI) and mortality and duration of inpatient stay. Outcomes were compared to previous data from our hospital site in 2015/2016. Results: Patients with severe cognitive impairment and systemic disease (Abbreviated Mental Test Score (AMTS) < 7 and American Society of Anesthesiologists (ASA) grade ≥ 3) showed significantly elevated NSSI risk, consistent across the study periods. Both periods demonstrated an increased risk of NSSI associated with admission from nursing homes. Despite the 2021/2022 cohort being notably older, NSSI risk decreased from 40.6% to 37.2% after implementing the orthogeriatric care model. NSSI risk was notably reduced for severe cognitive impairment (51.6% vs. 71%), and the *p*-value was 0.025. Average hospital stay decreased post-intervention (2.4 days shorter), with a notable reduction for NSSI patients (3.4 days shorter). Overall mortality rates were similar, although mortality due to infection was significantly reduced in 2021/2022 (44.4% vs. 93.3%), and the *p*-value was 0.003. Conclusion: The orthogeriatric liaison care model significantly decreased NSSI only in individuals with severe cognitive impairment and infection-associated mortality. This highlights the integral role of orthogeriatricians in the care of elderly hip fracture patients.

## 1. Introduction

Hip fractures contribute the highest morbidity, mortality, and economic cost of all fragility fractures [1,2,3]. The risk for hip fractures is intricate, involving general frailty, fall risk, and bone fragility. This vulnerability is reflected in the challenging outcomes observed in this patient group. The mortality and disability rate of elderly patients with hip fractures are high, and the death of hip fracture patients is closely associated with post-operative complications [4]. Due to the frailty observed in this patient group, the most commonly encountered complications are non-surgical, such as delirium, pneumonia, and thromboembolic events [5]. A meta-analysis of 8673 patients across England and Wales highlighted that non-surgical site infections, particularly chest infection and urinary tract infection, are the most common non-surgical post-operative complications among hip fracture patients, with an incidence of 6.3% and 5.0%, respectively. Other post-operative complications were less common, including deep vein thrombosis/pulmonary embolus (1.8%), cerebrovascular accident (0.6%), acute coronary syndrome (0.6%), and acute kidney injury (1.3%) [6]. Infections are not only common, but also a significant cause of morbidity and mortality following surgery and are reported among the leading causes of death in hip fracture patients [7]. Previous research highlights that the occurrence of infection within 30 days following hip fracture surgery is significantly linked to an elevated risk of mortality [8]. Beyond mortality, hospital-acquired infection is associated with increased length of hospital stay and cost to healthcare systems [9]. The incidence of hip fractures continues to rise, with incidence predicted to almost double from 2018 to 2050 [10]. It is therefore important to improve our understanding of factors that can increase the risk of post-operative complications, including infection, and the measures that can be put in place to prevent these. Against the backdrop of escalating global concerns regarding antimicrobial stewardship, the imperative to mitigate and manage infections in the peri-operative phase is of paramount importance. Understanding non-surgical site infections in hip fracture patients is therefore important not only to ensure patient safety but also to align with antimicrobial stewardship principles aimed at curtailing antimicrobial resistance and optimising healthcare resource allocation. The value of a multidisciplinary treatment approach is increasingly recognised to improve outcomes in hip fracture patients [11]. Orthogeriatric care constitutes a collaborative model that incorporates orthopaedic surgeons, geriatricians, and a multidisciplinary orthogeriatric team. In comparison to the conventional approach characterised by orthopaedic ownership with sporadic medical and geriatric input, orthogeriatric care has demonstrated superior outcomes. These include decreased mortality, shorter hospital stays, and a lower incidence of post-operative infections [1,12,13]. Zabawe et al. demonstrated in a review article that orthogeriatric liaison services were associated with decreased morbidity and mortality in individuals with dementia and hip fracture [14]. Importantly, Lisk et al., 2023 showed that orthogeriatric liaison services were associated with a significant decrease in the length of hospital stay, and the introduction of the service was also linked to savings of EUR 2.7 million per 1000 patients [15]. Several studies have indicated that orthogeriatric liaison services are associated with improvements in the length of hospital stay, decreased time to surgery, reduced early mortality, and improvements in early mobility [16,17,18,19,20,21]. The success of orthogeriatric services in acute hospitals or rehabilitation units depends on its multidisciplinary approach, including nutritional support [22,23]. There are many complications observed in the peri-operative period that necessitate vigilant oversight by the multidisciplinary team. Previous studies have shown a high incidence of deep vein thrombosis (DVT) post-operatively. Among 1209 cases of femoral neck fractures, 7.9% developed a DVT in the post-operative phase [24]. Niu et al. showed in 980 elderly patients with femoral neck fracture that the prevalence of DVT was 6.8%. [25]. DVT carries a high risk of mortality and morbidity and can potentially be on a similar scale to non-surgical site infection (NSSI). However, in this study, we compared our data with our previous published data, where infection was a significant contributor to mortality in hip fracture patients [26]. Despite the fact that DVT poses significant mortality and morbidity, it was not included in our previous study [26]. This not only underscores the importance of infection prevention and management within our trust but also offers valuable insights applicable to broader clinical settings. We aimed to identify improvements in clinical practice that can mitigate the burden of post-operative infections in hip fracture patients on a larger scale.

Over a 2-year period, our hospital underwent a transition in the model of care for hip fracture patients from a traditional to an orthogeriatric liaison model. Previously, patients were admitted to an orthopaedic trauma ward under an orthopaedic surgical team that remained primarily responsible for their care, with geriatrician input available on request. Now, patients are admitted under the orthopaedic team but receive regular orthogeriatric review on the orthopaedic ward. This involved daily orthogeriatric board rounds and thrice-weekly consultant-led orthogeriatric ward rounds, integrating multidisciplinary input into patient care.

This audit aimed to assess the impact of the orthogeriatric liaison care model within our hospital on patient outcomes such as incidence of hospital-associated infection, length of hospital stay, and inpatient mortality. As a secondary aim, this audit sought to identify whether certain pre-operative factors such as age, gender, Abbreviated Mental Test Score (AMTS), American Society of Anaesthesiologists (ASA), residence, time to theatre, and fracture type affected patient outcomes.

## 2. Methodology

### 2.1. Study Design

This was an audit conducted at Milton Keynes University Hospital, Milton Keynes, UK, designed in follow up to a previous study performed at our hospital in 2015/2016 [26].

### 2.2. Study Population

All patients who underwent surgery following hip fracture at a single hospital site over a 1-year period (1 July 2021–31 June 2022) were included. Femoral shaft, periprosthetic fracture, and patients managed non-operatively were excluded.

### 2.3. Data Collection

Data were retrieved from hospital patients’ records at Milton Keynes University Hospital. Data relating to patient demographics, treatment, and post-operative outcomes were collected, and all patient-identifiable data anonymised. Demographic variables included age, gender, pre-admission residence, pre-operative Abbreviated Mental Test Score (AMTS), American Society of Anaesthesiologists (ASA) grade, and fracture type. Patients were classified based on AMTS score into severe (AMTS < 7), moderate (AMTS 7–9), and no cognitive impairment (AMTS 10). Age was grouped into <65 years, 65–75 years, and >75 years. Furthermore, ASA score classified patients into five groups: no systemic disease (ASA 1), mild systemic disease (ASA 2), severe systemic disease (ASA 3), disease that is constantly a threat to life (ASA 4), and where the patient is not expected to survive the next 24 h (ASA 5). Data relating to treatment included date and time of admission, time to surgery, and type of treatment (surgical/nonsurgical). Time to surgery was classified into <36 h and >36 h. Post-operative outcomes included development of post-operative non-surgical site infection (NSSI) and type, length of inpatient stay, and inpatient mortality. Information relating to development of infection was retrieved from medical notes, nursing notes, discharge summaries, and investigation results. Patients with infection on admission were not included unless they subsequently developed an infection that was deemed different to that on admission. Patients who tested positive for COVID-19 were not included to enable comparison of infection rates with 2015/2016. Patients were deemed to have an infection if they met the following criteria, based on the Loeb criteria for diagnosing infection in long-term care settings: (1) symptoms of infection occurred post-operatively, for example, pyrexia > 37.9 °C or urinary symptoms; (2) positive clinical investigation such as urine dipstick, urine culture, sputum culture, or chest X-ray changes reported as likely infective; (3) treatment with antibiotics [27]. Specifically, the diagnosis of urinary tract infection was established based on urine microscopy and culture. Lower respiratory tract infection was diagnosed based on chest X-ray and blood results, including C-reactive protein and procalcitonin, and according to clinical assessment, further investigations were requested if needed (sputum culture, arterial blood gas analysis, or computed tomography scan of the chest). All patients received only one dose of prophylactic antibiotics intra-operatively, in line with National Institute for Health and Care Excellence (NICE) guidelines. Urinary catheterisation in the peri-operative period was typically avoided except in specific circumstances such as urinary retention. Early removal and trial without catheter were advocated in those who required catheterisation peri-operatively. Length of stay data were calculated from date of surgery to date of trust discharge. Date of surgery was used to facilitate a baseline to be taken across all patients. Factors that may influence hospital stay (hospital policy, availability of surgical theatres, implants, trained staff in peri-operative, and post-operative management) were excluded in order to decrease interference when comparison was performed between the two studies. Patients who died during their admission were excluded from length of stay calculations. The rehabilitation process was offered to all patients post-operatively and began on day one following surgery with an emphasis on early mobilisation and functional independence. Comprehensive rehabilitation is provided within the acute ward setting before discharge to the community. Throughout their stay on the acute ward, patients receive ongoing assessment, support, and education to optimise recovery and prepare for safe discharge. Discharge planning begins early and incorporates considerations such as home environment, support systems, and predicted ongoing care needs to facilitate transition to the community. Importantly, variability in responding to rehabilitation is expected in such cohorts of patients. As such, some patients will respond quickly and can be discharged earlier, and slow responders may exceed two weeks and be offered further rehabilitation in hospital or be discharged to rehabilitation centres in the community.

The primary outcome assessed was the incidence of post-operative non-surgical site infection. Secondary outcomes included length of hospital stay and inpatient mortality. Outcomes assessed were then compared to previous data collected from our hospital site in 2015/2016 to assess the effect of regular orthogeriatric input on these post-operative outcomes. Additionally, this audit aimed to identify the influence of certain pre-operative factors, including age, gender, AMTS, ASA, residence, time to theatre, and fracture type, on patient outcomes.

### 2.4. Statistical Analysis

IBM SPSS™ 23.0 was used for statistical analysis. Pearson Chi-squared (χ2) test determined significance between outcomes within patient sub-groups, which was deemed appropriate to compare categorical variables. The non-parametric Mann–Whitney U test was used to compare length of inpatient stay between patients with and without post-operative NSSI, as length of stay data were deemed to be non-normally distributed (Shapiro–Wilk). A *p*-value of <0.05 was considered statistically significant. Chi-squared test of independence was used in comparison between the two studies.

## 3. Results

### 3.1. Pre-Operative Measures

#### 3.1.1. Age, Gender, and Place of Residence

During our 1-year study period in 2021/2022 (Table 1), 258 patients underwent surgery for hip fracture. In 2015/2016, 207 patients over a 1-year period were included (Table 2). The majority of patients (74.8%) were >75 years of age on admission, 22.1% of patients were between 65 and 75 years, and only 3.1% were below 65 years of age. This is a notably older study population than the previous 2015/2016 study, where only 40.6% of patients were >75 years old on admission. The most common age group was 65–75 years (51.7%). The proportion of patients below 65 years of age was also higher previously (7.7%). In this study, 71.7% of the population were female, compared with 28.3% who were male. In 2015/2016, 66.2% of patients were female and 33.8% were male. In both studies, age and gender did not achieve significance as predictive measures of post-operative NSSI when tested using the chi-square test.

In this study, 86.8% of patients were admitted from their own homes, compared to 13.2% who were admitted from nursing homes. In 2015/2016, 78.7% of patients were admitted from their own homes, while 21.3% of patients were admitted from a nursing home. Both study periods saw an increased risk of post-operative infection in patients admitted from nursing homes compared to their own home. In 2021/2022, 50% of patients admitted from nursing homes developed an inpatient NSSI, while the corresponding figure for 2015/2016 was 54.5%. In the previous study period, this reached near significance (*p =* 0.059); however, in the current study period, this was no longer observed (*p =* 0.123).

#### 3.1.2. ASA and AMTS

In this study, the majority of patients (57.0%) were classified as ASA 3, followed by ASA 2 (24.8%). A smaller number of patients were classified as ASA 1 (3.9%) and ASA 4 (13.9%). Only one patient was categorised as ASA 5 as delay in surgery was deemed to lead to a significant increase in the threat to life. This was similar to the previous study period, where 56.0% were classified as ASA 3, 26.1% as ASA 2, 4.8% as ASA 1, and 13.4% as ASA 4. In both study periods, increasing ASA grade was shown to increase the risk of post-operative infection.

In the current study, 20% (2/10) of patients with no systemic disease (ASA 1) developed an NSSI. Meanwhile, 29.7% (19/64) of patients with mild systemic disease (ASA 2) developed an NSSI, 38.8% (57/147) of patients with severe systemic disease (ASA 3), and 50% (18/36) of patients with severe systemic disease that is a constant threat to life (ASA 4) developed an NSSI. A reduction in infection rates in patients with systemic disease was observed in our study population compared to previously. In 2015/2016, infection rates were 59.1% in ASA 4, 46.6% in ASA 3, and 25.9% in ASA 2 groups.

In the current study population, 24.8% (64/258) of patients had severe cognitive impairment (AMTS < 7), and 31.4% (81/258) of patients had moderate cognitive impairment (AMTS 7–9). Most patients, 42.6% (110/258), had no cognitive impairment (AMTS 10). In addition, three patients were not assigned AMTS before surgery, with no reason given. This was similar to the 2015/2016 study period, where 30% of patients had severe cognitive impairment, and 25.6% of patients had moderate cognitive impairment. Again, the majority of patients had no cognitive impairment (41.6%). There was a significant reduction in the risk of post-operative infection with normal pre-operative cognitive function (*p* ≤ 0.05). Furthermore, 51.6% (33/64) of patients with severe cognitive impairment developed an NSSI (*p* = 0.017). This was more than double the proportion of patients with no cognitive impairment who developed a post-operative infection (23.6%, 26/110). In patients with moderate cognitive impairment, 45.7% (37/81) developed an NSSI following their operation. Data from both study periods demonstrated patients with severe cognitive impairment are at an increased risk of NSSI. Notably, the incidence of NSSI in patients with severe cognitive impairment fell in this study period from 71.0% in 2015/2016, representing a relative risk reduction of 27.3%. Infection rates for patients with moderate cognitive impairment were 34.0% and 25.6% in patients with no cognitive impairment previously. The chi-square test showed a significantly increased risk of post-operative infection in patients with both severe cognitive impairment and severe systemic disease (AMTS < 7 and ASA 3 or 4) in the current study period (51.7% (31/60) developed an NSSI following their operation (*p* = 0.02). However, when we compared our data with data from 2016, a significant decrease in NSSI in patients with severe cognitive impairment was noted (*p*-value = 0.025) (Table 3).

#### 3.1.3. Type of Fracture and Time to Theatre

In the current study period, the most common fracture types were intertrochanteric (121/258, 46.9%) and displaced intra-capsular fractures (105/258, 40.7%). Undisplaced intra-capsular fractures accounted for 11.2% (29/258), while subtrochanteric fractures represented 1.2% (3/258) of our population. This was similar to the previous study population; intertrochanteric fractures (44.4%) and displaced intra-capsular fractures (42.5%) were again the most common. Undisplaced intra-capsular fractures were less frequent (6.8%), and sub-trochanteric fractures were more frequent (6.3%). In both periods, the majority of patients received surgery within 36 h of admission: 185/258 (71.7%) in 2021/2022 and 160/207 (77.3%) in 2015/2016.

Time to theatre and fracture type did not achieve significance as predictive measures of post-operative NSSI when using the chi-square test in both study periods.

### 3.2. Post-Operative Outcomes

#### 3.2.1. Development of Infection

Overall, 37.2% (96/258) of patients developed an NSSI following hip fracture surgery. The most common inpatient non-surgical site infections were lower respiratory tract infections (43/96, 44.8%) and urinary tract infections (39/96, 40.6%). Additionally, 6 out of 96 (6.3%) patients developed more than one type of infection (mixed), and 7.3% (7/96) of infections were of an unknown source. There was also one patient (1%) who developed cellulitis, which was not associated with the wound site. A lower incidence of inpatient NSSI was observed in the current study period. Previously, with the traditional model of care, 40.6% (84/207) of patients developed NSSI (Table 3). The type and proportion of NSSI seen in both study periods did not change significantly.

#### 3.2.2. Length of Stay

During the study period, 18 patients died as inpatients and were thus excluded from length of stay calculations. The average length of stay following surgery was 21.5 days (SD = 16 days) (n = 240). Patients who developed an NSSI stayed an average of 28.2 days (SD = 16.2 days) in hospital. This was significantly longer than those who did not develop an NSSI, who stayed for an average of 18 days (SD = 14.6 days, *p* < 0.001). In both periods, the development of NSSI resulted in an increased length of stay. Development of infection increased length of stay by an average of 13.1 days (from 18.5 days to 31.6 days) in 2015/2016 and 10.2 days (from 18 to 28.2 days) in 2021/2022. Notably, the length of hospital stay was, on average, 2.4 days shorter following the implementation of an orthogeriatric liaison care model compared to the previous traditional model of care (21.5 days vs. 23.9 days). Length of stay was also 3.4 days shorter for patients who developed an NSSI with the orthogeriatric care model (28.2 days vs. 31.6 days) (Table 3).

#### 3.2.3. Mortality

Overall, 7% (18/258) of patients died during their hospital stay. Of these, eight (44.4%) had infective causes, all of which were from lower respiratory tract infections (LRTI). Among these, two patients developed more than one infection during their hospital stay. Other causes included pulmonary embolism (1/18), decompensated liver failure (1/18), renal failure on a background of chronic kidney disease (2/18), and perforated sigmoid diverticulum (1/18). In addition, five patients died due to severe frailty, as stated on their death certificate. The mortality rate was very similar between the two cohorts (Table 3). However, the proportion of patients who developed an infection and subsequently died because of that infection was significantly higher in 2015/2016, *p*-value = 0.003. In 2015/2016, 15 (7.2%) patients died as an inpatient. Of these, the majority (14/15; 93.3%) had infection as the main cause of death.

## 4. Discussion

Regular orthogeriatric input has been demonstrated to be associated with significant improvement in decreasing length of hospital stay, reduction of financial burden, decreased time to operation, reduced mortality, early mortality, and improvement in early mobility [15,16,17,18,19,20,21]. This study demonstrates that the implementation of regular orthogeriatric review is associated with a significant decrease in non-surgical site infections (NSSIs) only in individuals with severe cognitive impairment and infection-associated mortality. Our study also showed a reduction in the length of stay, but not to a significant level. This emphasises the importance of conducting a comprehensive geriatric assessment and proactive identification and management of risk factors that lead to high mortality and morbidity. It is also important to note that these improvements in post-operative outcomes in the current study period have been observed in a significantly older population. The prevention and management of infections in elderly patients with hip fractures can be intricate and necessitate vigilant oversight by ortho-geriatricians. This complexity can arise from various factors such as increasing age, frailty, cognitive impairment, and comorbidity, as described here and in previous literature. Increasing age is a well-established risk factor for post-operative morbidity and mortality and increases the risk of frailty [28]. Frailty has also been demonstrated as a significant predictor of adverse post-operative outcomes, including increased mortality, post-operative complications, including NSSI, and increased length of hospital stay across various disciplines [29,30,31], including hip fracture surgery [32,33]. Previous studies demonstrate that severe cognitive impairment increases the risk of post-operative infection following hip fracture [20], particularly respiratory and urinary tract infections [34,35]. Here, the implementation of an orthogeriatric liaison model reduced infection frequency in this group. A decline in NSSI frequency was also observed in patients with severe systemic disease, such that the trend of increasing ASA grade no longer met significance for predicting the development of NSSI in the current study population. Increasing ASA grade has previously been shown to predict post-operative morbidity and mortality in hip fractures [7,20,28,36,37,38]. NSSI also decreased in patients admitted from nursing homes, which was no longer near significant in the current study period. Patients from residential homes have also been demonstrated to be at a higher risk of mortality and post-operative complications [20,38] due to the comorbidity and frailty associated with admittance into nursing homes [39].

Importantly, this audit has also shown that implementing orthogeriatric care models can reduce the length of hospital stay, which supports the findings from previous literature [1,13,40]. Here, the introduction of orthogeriatric liaison care resulted in a reduction in hospital stay by an average of 2.4 days, resulting in an estimated saving of EUR 1420 [41]. This may reflect the efficiency gained through coordinated and targeted interventions. Orthogeriatric expertise enables the identification of potential barriers to recovery and the tailoring of rehabilitation plans to optimise functional outcomes. Moreover, the emphasis on the proactive management of potential complications such as pain, delirium, hypovolaemia, and anaemia can help ensure successful recovery, reducing the need for extended hospitalisation. Early and coordinated discharge planning, lower complication rates, and timely management of complications associated with orthogeriatric care may lead to a shorter length of inpatient stay. This audit also demonstrated a significant decline in infection-related mortality. This may be explained by improved monitoring of patients, earlier detection of complications, and more timely intervention through regular orthogeriatric review [42].

Previous studies demonstrate that integrated orthogeriatric treatment is associated with a reduction in hospital mortality and long-term mortality rates [43,44,45,46,47,48]. A meta-analysis including 18 studies (9094 patients) concluded that combined orthopaedic and geriatrician care reduced mortality [13]. A more recent meta-analysis showed a 28% lower risk of inpatient mortality with orthogeriatric care [1]. However, in our audit, introducing an orthogeriatric care model did not significantly reduce overall mortality. A potential explanation for this may be the significantly older population in our cohort, with more complex care needs and frailty [48]. A more accurate estimation of the impact of orthogeriatric liaison care on mortality may require a larger sample size and recruitment from different centres.

Perhaps a future prospective study may allow for the assessment of different variables in relation to morbidity and mortality with a longer duration of assessment and use of logistic regression analysis. Other limitations include the use of a historical comparison group and the reliance on the accuracy of data collated for the national hip fracture database. The AMTS and ASA grade, although widely used, have their limitations, including an inherent risk of assessor bias. Perhaps larger studies with recruitment from different centres may confirm the observations reported here, and more details can be gained with community follow up. Future studies may also examine the impact of additional variables, including thromboembolic events. The influence of surgical site infections (SSI) and their impact on NSSI would be important to assess. Additionally, determining whether early occurrences of SSI may be the driving factor for NSSI should be the focus of investigation. Using different variables may allow for more precision in the assessment of morbidity and mortality.

## 5. Conclusions

The contribution of orthogeriatricians in the management of hip fracture patients significantly decreased infection only in individuals with severe cognitive impairment and infection-associated mortality. Therefore, orthogeriatric care represents an integral part of the orthopaedic service. The input from the multidisciplinary team and regular orthogeriatric review can help optimise the medical management of these patients alongside surgical management. This can help to improve the length of stay, decrease complication rates, and reduce in-hospital mortality, particularly in relation to infection, as well as improve the quality of care and reduce healthcare costs.

## Figures and Tables

**Table 1:** The number of patients (and percentage of patients) who did and who did not develop NSSI after the implementation of an orthogeriatric liaison model of care, 2021/2022.

2021/2022		Number of Patients (% of Each Group)		
	Total	Yes NSSI	No NSSI	*p*-Value
Overall NSSI	258	96 (37.2%)	162 (62.8%)	
AMTS				
<7	64 (24.8%)	33 (51.6%)	31(48.4%)	0.017 *
7–9	81 (31.4%)	37 (45.7%)	44 (54.3%)	0.114
10	110 (42.6%)	26 (23.6%)	84 (76.4%)	0.003 *
Not assigned before surgery	3 (1.20%)	0 (0.00%)	3 (100.0%)	
ASA				
1	10 (3.90%)	2 (20.0%)	8 (80.0%)	0.260
2	64 (24.8%)	19 (29.7%)	45 (70.3%)	0.214
3	147 (57.0%)	57 (38.8%)	90 (61.2%)	0.693
4	36 (13.9%)	18 (50.0%)	18 (50.0%)	0.112
5	1 (0.40%)	0 (0.00%)	1 (100.0%)	
Age				
<65 years	8 (3.10%)	2 (25.0%)	6 (75.0%)	0.475
65–75 years	57 (22.1%)	13 (22.8%)	44 (77.2%)	0.025 *
>75 years	193 (74.8%)	81 (42.0%)	112 (58.0%)	0.170
Residence				
Nursing home	34 (13.2%)	17 (50.0%)	17 (50.0%)	0.123
Own home	224 (86.8%)	79 (35.3%)	145 (64.7%)	0.550
Gender				
Male	73 (28.3%)	28 (38.4%)	45 (61.6%)	0.838
Female	185 (71.7%)	68 (36.8%)	117 (63.2%)	0.901
Fracture type				
Intra-capsular (displaced)	105 (40.7%)	42 (40.0%)	63 (60.0%)	0.553
Intra-capsular (un-displaced)	29 (11.2%)	8 (27.6%)	21 (72.4%)	0.284
Intertrochanteric	121 (46.9%)	44 (36.4%)	77 (63.6%)	0.849
Sub-trochanteric	3 (1.20%)	2 (66.7%)	1 (33.3%)	0.890
Time to theatre				
<36 h	185 (71.7%)	67 (36.2%)	118 (63.8%)	0.782
>36 h	73 (28.3%)	29 (39.7%)	44 (60.3%)	0.655

AMTS = abbreviated mental test score; ASA = American Society of Anaesthesiologists. * = *p* < 0.05. *p*-values are represented for the risk of infection for each patient group.

**Table 2:** The number of patients (and percentage of patients) who did and who did not develop NSSI when a traditional model of care was in place in 2015/2016. Predictors of infective outcomes following hip fracture, 2016, by permission of Gerontology and Geriatric Medicine [26].

2015/2016	Number of Patients (% of Each Group)			
	Total	Inpatient NSSI	No NSSI	*p*-Value
Overall NSSI	207	84 (40.6%)	123 (59.4%)	
AMTS				
<7	62 (30%)	44 (71.0%)	18 (29.0%)	<0.001 *
7–9	53 (25.6%)	18 (34.0%)	35 (66.0%)	0.327
10	86 (41.6%)	22 (25.6%)	64 (74.4%)	0.005 *
ASA				
1	10 (4.8%)	0 (0.0%)	10 (100.0%)	0.009 *
2	54 (26.1%)	14 (25.9%)	40 (74.1%)	0.028 *
3	116 (56.0%)	54 (46.6%)	62 (53.4%)	0.19
4	27 (13.4%)	16 (59.3%)	11 (40.7%)	0.048 *
Age				
<65 years	16 (7.7%)	3 (18.8%)	13 (81.3%)	0.075
65–75 years	107 (51.7%)	39 (36.5%)	68 (63.6%)	0.384
>75 years	84 (40.6%)	42 (50.0%)	42 (50.0%)	0.079
Residence				
Nursing home	44 (21.3%)	24 (54.5%)	20 (45.5%)	0.059
Own home	163 (78.7%)	60 (36.8%)	103 (63.2%)	0.327
Gender				
Male	70 (33.8%)	26 (37.1%)	44 (62.9%)	0.558
Female	137 (66.2%)	58 (42.3%)	79 (57.7%)	0.676
Type of fracture				
Intra-capsular (displaced)	88 (42.5%)	36 (40.9%)	52 (59.1%)	0.95
Intra-capsular (undisplaced)	14 (6.80%)	3 (21.4%)	11 (78.6%)	0.144
Intertrochanteric	92 (44.4%)	38 (41.3%)	54 (58.7%)	0.887
Sub-trochanteric	13 (6.30%)	7 (53.8%)	6 (46.2%)	0.33
Time to theatre				
<36 h	160 (77.3%)	63 (39.4%)	97 (60.6%)	0.756
>36 h	47 (22.7%)	21 (44.7%)	26 (55.3%)	0.567

AMTS = abbreviated mental test score; ASA = American Society of Anaesthesiologists. * = *p* < 0.05. *p*-values are represented for the risk of infection for each patient group. This content is not covered by the terms of the Creative Commons licence of this publication. For permission to reuse, please contact the rights holder.

**Table 3:** Comparison of outcomes between the two study periods.

	2015/2016	2021/2022	*p*-Value
Percentage of patients who developed NSSI			
NSSI	40.6%	37.2%	0.458
Non-NSSI	59.4%	62.8%	
AMTS			
<7	71.0%	51.6%	0.025 *
7–9	34.0%	45.7%	0.178
10	25.6%	23.6%	0.189
ASA			
1	0.0%	20.0%	0.136
2	25.9%	29.7%	0.650
3	46.6%	38.8%	0.205
4	59.3%	50.0%	0.466
Age			
<65 years	18.8%	25.0%	0.722
65–75 years	36.5%	22.8%	0.075
>75 years	50.0%	42.0%	0.216
Residence			
Nursing home	54.5%	50.0%	0.690
Own home	36.8%	35.3%	0.755
Gender			
Male	37.1%	38.4%	0.881
Female	42.3%	36.8%	0.310
Type of fracture			
Intra-capsular (displaced)	40.9%	40.0%	0.898
Intra-capsular (undisplaced)	21.4%	27.6%	0.665
Intertrochanteric	41.3%	36.4%	0.463
Sub-trochanteric	53.8%	66.7%	0.687
Time to theatre			
<36 h	39.4%	36.2%	0.546
>36 h	44.7%	39.7%	0.591
Mortality			
Overall	7.2%	7.0%	0.910
As result of infection	93.3%	44.4%	0.003 *
Length of stay (days)			
Overall	23.9 days	21.5 days	0.651
atients who developed NSSI	31.6 days	28.2 days	0.287
Patients who did not develop NSSI	18.5 days	17.7 days	0.582

NSSI = non-surgical site infection; AMTS = abbreviated mental test score; ASA = American Society of Anaesthesiologists. * = *p* < 0.05. *p*-values are represented for the risk of infection for each patient group. The table included data for NSSI as this facilitates comparison and readers are recommended to see Table 1 and Table 2 for data of patients without NSSI.

## Data Availability

Data will be available on request.
